# Microbiome dataset analysis from a shrimp pond in Ninh Thuan, Vietnam using shotgun metagenomics

**DOI:** 10.1016/j.dib.2020.105731

**Published:** 2020-05-21

**Authors:** Lan-Anh Le, Thien-Phuc Nguyen-Hoang, Van-Phuc Huynh, Tan-Huy Nguyen, Thuy-Vy Nguyen, Thuy-Duong Ho-Huynh

**Affiliations:** a Khoa Thuong Biotechnology Company Limited, No 17, Street 4, Phong Phu, Binh Chanh, Ho Chi Minh, Vietnam; b Department of Genetics, Faculty of Biology and Biotechnology, University of Science, VNU-HCM, 227 Nguyen Van Cu Street, Ward 4, District 5, Ho Chi Minh, Vietnam; c Research Center for Genetics and Reproductive Health, School of Medicine, VNU-HCM, 6 Residential area, Linh Trung, Thu Duc, Ho Chi Minh, Vietnam

**Keywords:** Metagenomics, Shotgun sequencing, Oxford Nanopore MinION sequencing, Aquaculture, Shrimp pond

## Abstract

Vietnam is one of the top shrimp producing and exporting countries in the world [1]. However, viral and bacterial epidemic diseases cause severe damages to shrimp farming, resulting in millions of US dollars losses annually [2]. Furthermore, inappropriate use of antibiotics in shrimp rearing lead to increased emergence of drug resistant pathogens [3]. Current practices for water quality control, mostly based on chemical and physical parameters; neglected biological criteria necessary for maintaining pond health.

Ninh Thuan is a region situated in the South Central Coast of Vietnam. Due to its geographic location, a large part of this region is dedicated to shrimp (*Litopenaeus vannamei)* post-larvae production and rearing. This article presents a microbiome dataset from two water samples collected in a shrimp rearing pond in Ninh Thuan. We used Oxford Nanopore Technologies (ONT) for metagenomic sequencing of the samples to characterize microbial communities and antibiotic resistance profiles. The metagenome dataset generated will provide an understanding and comparison framework of the microbial diversity and functionality among shrimp ponds with potential application in health management and shrimp rearing industry.

Specifications Table

**Table:** 

Subject	Applied Microbiology and Biotechnology
Specific subject area	Metagenomics
Type of data	Figures and Fastq files
How data were acquired	MinION (ONT)
Data format	Raw and analyzed
Parameters for data collection	Water samples collected from a shrimp pond at 0.3-m depth
Description of data collection	Total DNA was extracted from water samples, and shotgun metagenomic sequencing was performed using ONT MinION platform
Data source location	Village/Town/City: Phuoc Dinh, Thuan Nam, Ninh Thuan Country: Vietnam Latitude and longtitude coordinates for collected samples: 11°24′46.6"N, 108°58′19.0"E and 11°24′46.4"N, 108°58′17.0"E
Data accessibility	Data is available at the NCBI with Bioproject PRJNA552940 and SRA accession numbers SRR9648445 (https://trace.ncbi.nlm.nih.gov/Traces/sra/?run=SRR9648445) and SRR9648446 (https://trace.ncbi.nlm.nih.gov/Traces/sra/?run=SRR9648446).

## Value of the Data

• The analysis revealed an insight of microbiome present in water of a shrimp rearing pond in Vietnam.
• Data obtained from this article provided the scientific community with microbial taxonomic profiles and functional characteristics of a shrimp pond using metagenomics.
• Data can be used for the comparison of taxonomic and functional profiles among shrimp ponds with different geographic distribution.
• Data can serve future analyses of the relationship between microorganism profiles of shrimp pond water and risks of disease outbreak as well as health threats due to antibiotic resistance genes spread.

## 1 Data Description

**Fig. 1:**
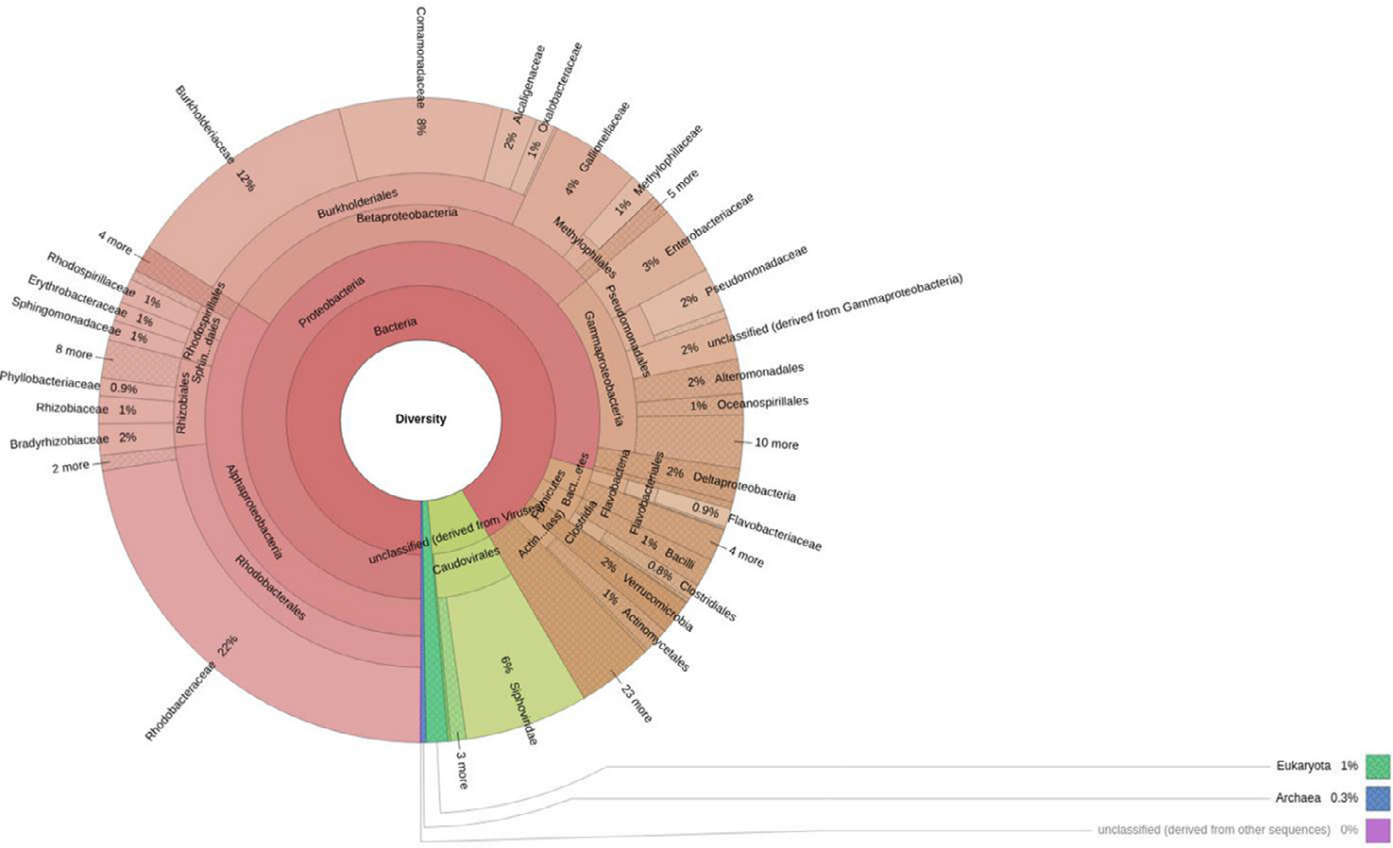
Taxonomic metagenomic profile of a water sample from shrimp pond - Ninh Thuan, Vietnam. The most abundant domain was bacteria (94.28%), followed by viruses (3.68%), eukaryota (1.77%), and archaea (0.08%). Among bacteria, phylum observed in a descending order of magnitude included Proteobacteria, Actinobacteria, Bacteroidetes, Firmicutes, Cyanobacteria. Of the 146 bacterial orders detected, the most abundant were Burkholderiaceae (21.07%), Rhodobacteraceae (17.85%) and Gallionellaceae (6.64%). Moreover, a total of 252 families and 515 genus was identified.

**Fig. 2:**
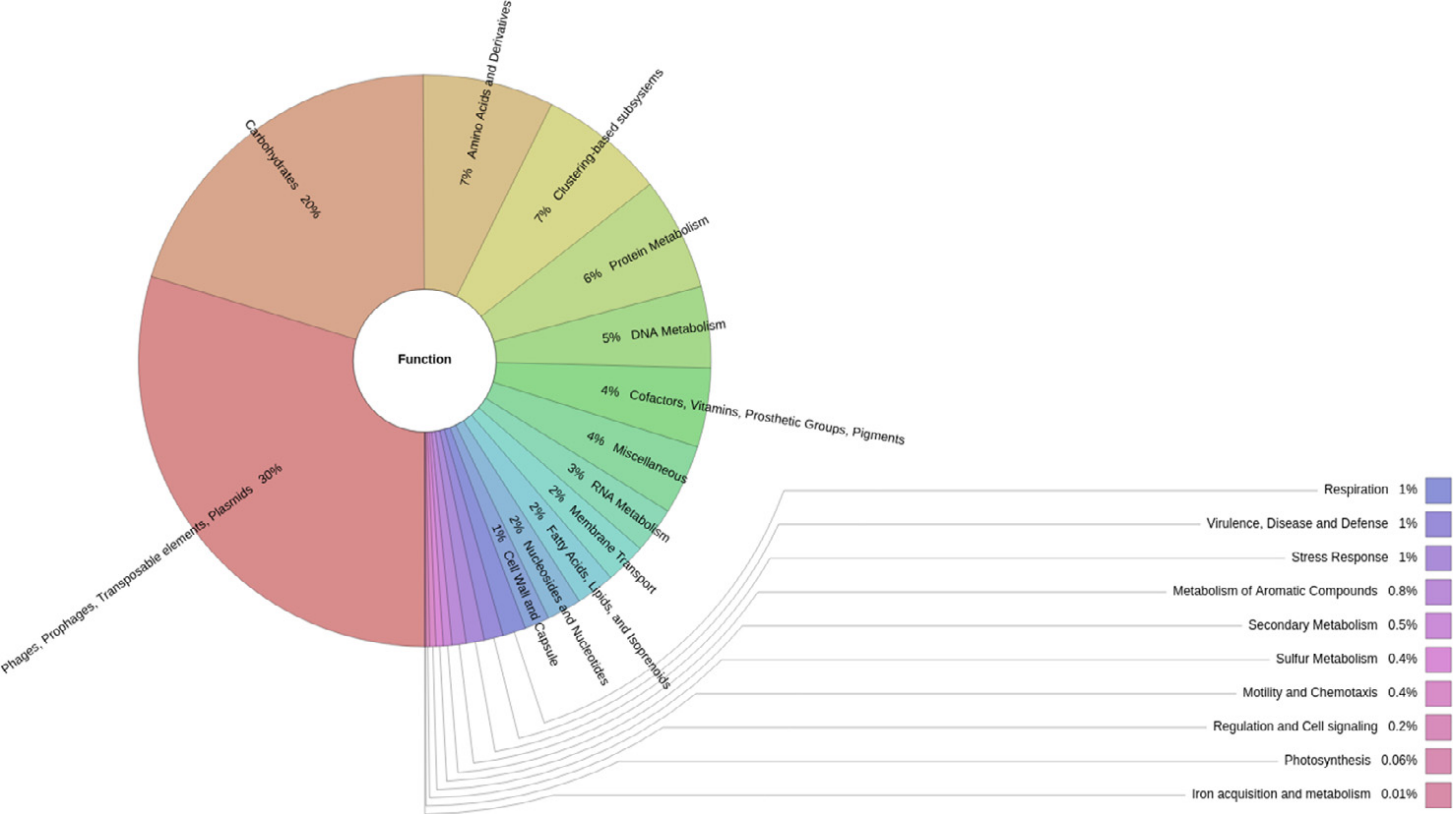
Functional metagenomic profile of a water sample from shrimp pond - Ninh Thuan, Vietnam. The most abundant function corresponded to phages, transposable elements and plasmids (29.97%); followed by clustering-based subsystems (14.02%), carbohydrates (8.96%), amino acids and derivatives (8.51%), protein metabolism (6.23%); and other categories (32.78%).

The data in this dataset described the taxonomic and functional profiles of two metagenomic samples from a shrimp pond in Ninh Thuan, Vietnam. We performed shotgun sequencing on ONT MinION platform and analyzed data using MG-RAST server and KmerResistance. A total of 28,837 (sample 1) and 24,434 (sample 2) reads were classified out of 60,944 (sample 1) and 51,784 (sample 2) reads sequenced. Data was presented as taxonomic and functional profiles in Figs. 1 and 2, respectively. Additionally, antimicrobial resistance determinants against beta-lactamase, aminoglycosides, and sulphonamide were observed in both samples from the same template acquired from *Pseudomonas aeruginosa (aadA2b, sul1_2_U12338), Klebsiella pneumoniae (sul1_EU780013), Vibrio cholerae strain MO10 (sul2_2_AY034138)* (Supplementary Table 1).

## 2 Experimental Design, Materials, and Methods

### 2.1 Sample collection and DNA extraction

Two water samples were collected at two different sites, approximately at 0.3-m depth, from a shrimp rearing pond located at Ninh Thuan Province, Vietnam in March 2019. Samples were delivered to the laboratory on ice in 9 hours. Filtered samples (50 ml) were submitted to DNA extraction using the QIAamp Blood DNA mini kit (Qiagen, Germany). DNA quality and concentration were assessed by spectrophotometer and Qubit dsDNA HS assay kit (Life Technologies).

### 2.2 Metagenome library preparation and sequencing

Sequencing library was prepared using 1D Rapid PCR Barcoding Kit (SQK-RPK004) according to the manufacturer's instructions. PrimeSTAR GXL DNA polymerase (Takara, Japan) was utilized for amplification. The two libraries were sequenced for 14 hours on MinION device with FLO-MIN106 (R9.4) flowcell.

### 2.3 Taxonomic and functional analysis

Reads were preprocessed by base-calling and demultiplexing using Guppy v.2.3.5 (ONT). Those reads were quality-checked, filtered to remove sequence reads shorter than 500 bp and reads with an average quality score less than 7 using NanoFit v.2.5.0 [4], and MinKnow v.19.10.1 (ONT). For taxonomic identification and functional prediction, the filtered sequences were submitted to MG-RAST online server [5] using the M5nr database and subsystems based annotation with default setting, respectively. Cut-offs included a maximum E-value of 1 × 10^−5^, and a minimum alignment length of 15 were used. KmerResistance v.2.2 [6] was used for antibiotic resistance genes analysis with 90% identity and 10% depth corr.

## Declaration of Competing Interest

There is no conflict of interest to declare.

## Appendix Supplementary materials

Image, application 1

Image, application 2
